# Supplementary material for: A behavior-environment information loop drives sensory navigation
Source: ArXiv. 2026 Jul 28:arXiv:2607.26295v1. Preprint. [Version 1] (PMC13618432)
Supplement: Supplementary file 1 [file NIHPP2607.26295v1-supplement-1.pdf]

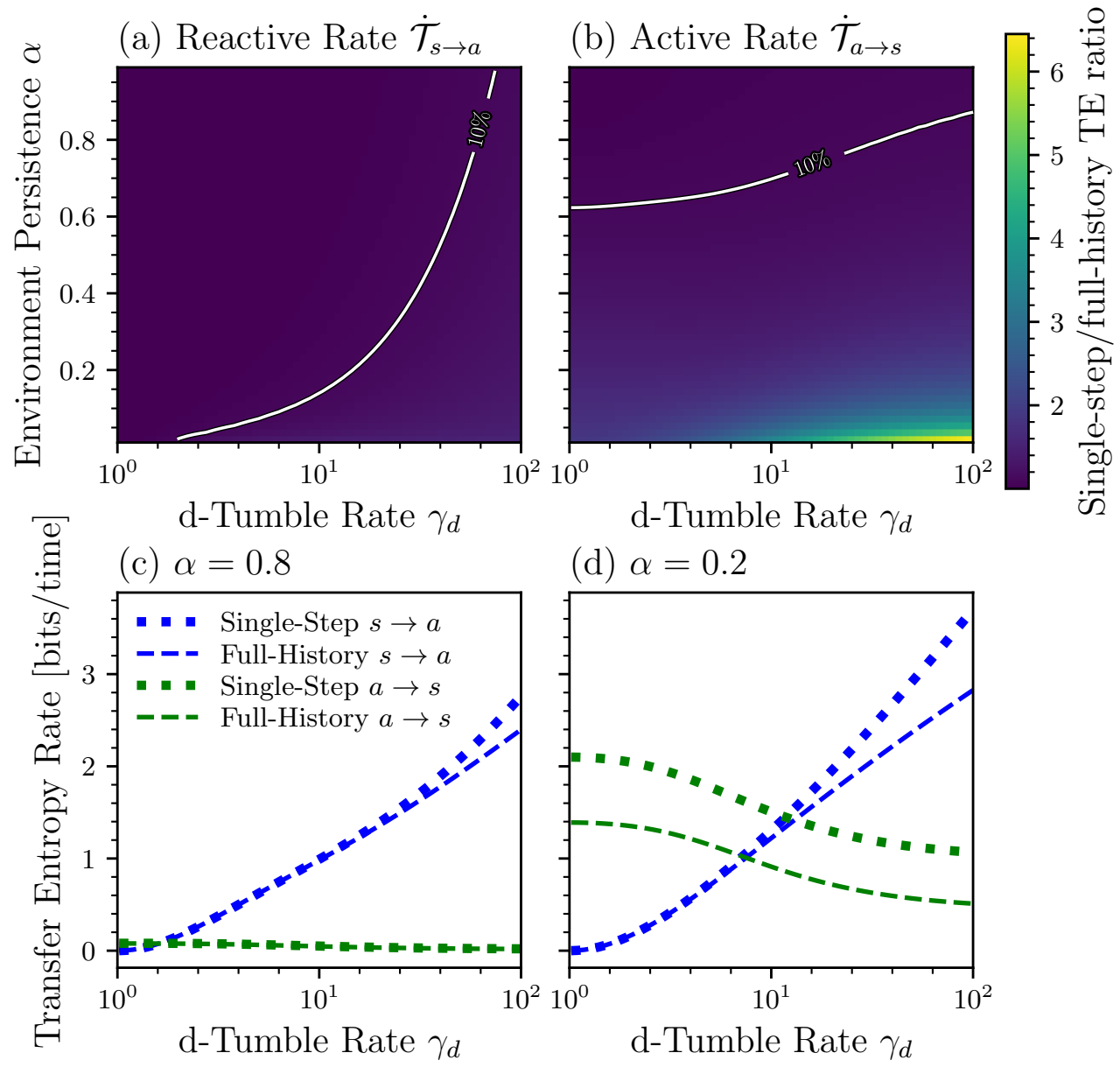

Supplementary Figure 1. Heatmaps showing the ratio between single-step and full-history transfer entropy rates for both the reactive (a) and active (b) rates, as functions of γ_d and α. We take $\kappa = 1$, $\lambda = 10$, and $\gamma_u = \kappa/\gamma_d$. (c) and (d) Cross-sections showing the single-step and full-history transfer entropy rates as functions of γ_d for $\alpha = 0.8$ (c) and $\alpha = 0.2$ (d). All transfer entropy rates shown are in bits.

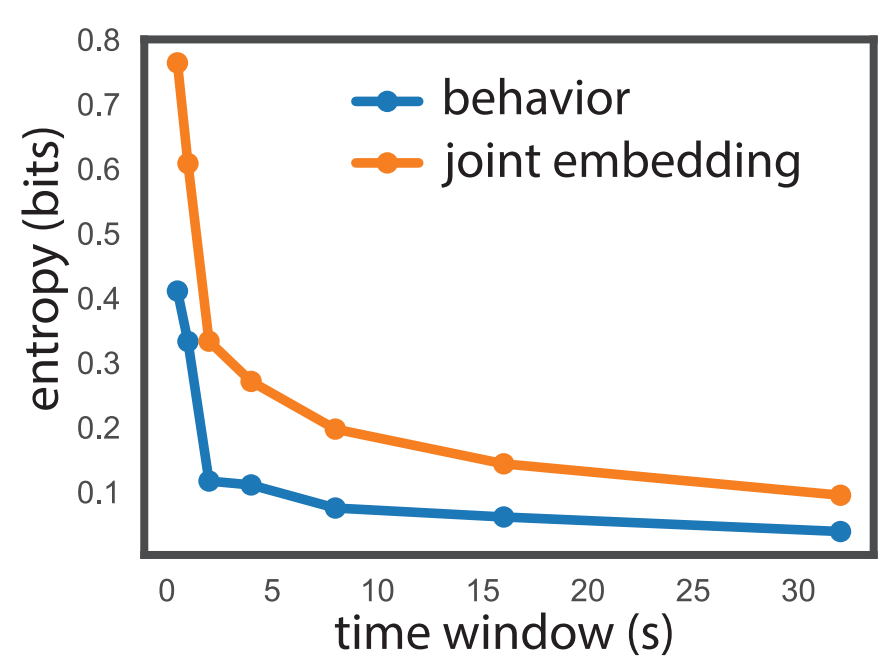

Supplementary Figure 2. Time delayed embedding to find consistent state transition models. As a function of the time window size, we build two-state Markov models fitted to data and computed the steady-state entropy rate. Models for behavior-only and joint embedding with sensory signal are shown. This example data is from bacteria chemotaxis data, showing ~ 2 s embedding gives a consistent state-space description.

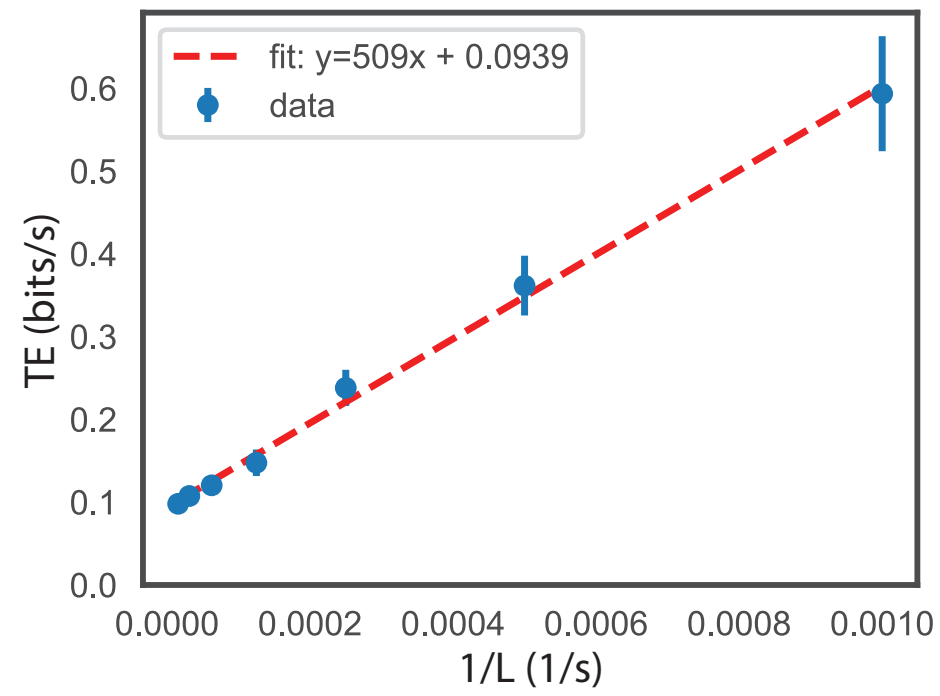

Supplementary Figure 3. Finite size correction for information estimation. We follow previous methods [30] to correct for finite size over-estimation of information content by extrapolating non-zero transfer entropy (TE) rate at infinite data length (L). Here the extrapolated TE rate is ≈ 0.09 bits/s. This example is for bacteria chemotaxis, with error bars showing 100 repeated subsampling.

Supplemental Information: Analytical Derivations for Φ and Full-History Transfer Entropy Rates

I. A GENERAL PROOF FOR THE LOW-INFORMATION SCALING OF THE CHEMOTACTIC INDEX

Here we prove that the functional form of the low-information expansion of Δ, which we call the metric Φ, will arise for a very general class of chemotaxis models. Consider a chemotaxis system with bipartite degrees of freedom s_t and a_t which each take a set of discrete values. Because of the bipartite decomposition, transition rates are only non-zero when either a changes states from a to a' at fixed s (with rate $w^s_{a'a}$) or s changes states from s to s' at fixed a (with rate $w^{s's}_a$).

We now define the coarse-grained transition rates

$$\bar{w}_{a'a} \equiv \sum_s p(s|a) w^s_{a'a}, \tag{1a}$$

$$\bar{w}^{s's} \equiv \sum_a p(a|s) w^{s's}_a. \tag{1b}$$

These in turn let us define the single-step transfer entropy rates,

$$\dot{T}_{s\to a} \equiv \left\langle \sum_{a'} w^s_{a'a} \ln \frac{w^s_{a'a}}{\bar{w}_{a'a}} \right\rangle_{p(a,s)}, \tag{2a}$$

$$\dot{T}_{a\to s} \equiv \left\langle \sum_{s'} w^{s's}_a \ln \frac{w^{s's}_a}{\bar{w}^{s's}} \right\rangle_{p(a,s)}. \tag{2b}$$

Now, suppose there exist parameters x and y such that

$$w^s_{a'a} = w^0_{a'a}\left[1 + x\, f^{(1)}_y(s,a,a') + x^2\, f^{(2)}_y(s,a,a') + \mathcal{O}\left(x^3\right)\right], \tag{3a}$$

$$w^{s's}_a = w^{s's}_0\left[1 + y\, g^{(1)}_x(a,s,s') + y^2\, g^{(2)}_x(a,s,s') + \mathcal{O}\left(y^3\right)\right]. \tag{3b}$$

In this way, x and y orthogonally control state-dependent differentiation of the transition rates from their coarse-grained average values, since when $x=0$ we have $w^s_{a'a} = \bar{w}_{a'a}$ for all s, and when $y=0$ we have $w^{s's}_a = \bar{w}^{s's}$ for all a. Thus clearly $\dot{T}_{s\to a} = 0$ when $x=0$, and $\dot{T}_{a\to s} = 0$ when $y=0$. We will now walk through the full derivation step-by-step for $\dot{T}_{s\to a}$, with the equivalent result for other transfer entropy rate following by symmetry,

Probabilities

Consider the case where y is finite, and x is small. We can expand the probability distribution around $x=0$ as

$$p_{x,y}(s,a) = p^{(0)}_y(s,a) + x\, p^{(1)}_y(s,a) + \mathcal{O}(x^2), \tag{4}$$

where $\sum_{s,a} p^{(1)}_y(s,a) = 0$ to ensure normalization for all x. Defining $u^{(i)}_y(s) = \sum_a p^{(i)}_y(a,s)$ and $v^{(i)}_y(a) = \sum_s p^{(i)}_y(a,s)$, we can then write the marginal probability expansions as

$$p_{x,y}(s) = u^{(0)}_y(s) + x\, u^{(1)}_y(s) + \mathcal{O}\left(x^2\right), \tag{5a}$$

$$p_{x,y}(a) = v^{(0)}_y(a) + x\, v^{(1)}_y(a) + \mathcal{O}\left(x^2\right), \tag{5b}$$

where $u^{(1)}_y(s)$ and $v^{(1)}_y(a)$ both sum to 0. Finally, the conditional distribution $p(s|a)$ is then

$$p_{x,y}(s|a) = p^{(0)}_y(s|a) + x \underbrace{\left[\frac{p^{(1)}_y(s,a) - p^{(0)}_y(s|a) v^{(1)}_y(a)}{v^{(0)}_y(a)}\right]}_{p^{(1)}_y(s|a)} + \mathcal{O}\left(x^2\right). \tag{6}$$

Here we have defined $p^{(0)}_y(s|a) = p^{(0)}_y(s,a)/v^{(0)}_y(a)$, and $p^{(1)}_y(s|a)$, which must satisfy $\sum_s p^{(1)}_y(s|a) = 0$ to ensure normalization. It will prove useful later to note the relation

$$p^{(1)}_y(s,a) = p^{(1)}_y(s|a) v^{(0)}_y(a) + p^{(0)}_y(s|a) v^{(1)}_y(a). \tag{7}$$

A. Transfer Entropy Rates

We now proceed to evaluate the transfer entropy rates, first beginning with the coarse-grained transition rates:

$$\begin{aligned}\bar{w}_{a'a} &= \sum_s \left[p_y^{(0)}(s|a) + x\, p_y^{(1)}(s|a) + \mathcal{O}(x^2)\right] w^0_{a'a} \left[1 + x\, f_y^{(1)}(s,a,a') + x^2\, f_y^{(2)}(s,a,a') + \mathcal{O}\left(x^3\right)\right] \\ &= w^0_{a'a}\left[1 + x \underbrace{\sum_s [p_y^{(0)}(s|a) f_y^{(1)}(s,a,a')}_{\bar{f}_y^{(1)}(a,a')} + x^2 \underbrace{\sum_s \left(p_y^{(0)}(s|a) f_y^{(2)}(s,a,a') + p_y^{(1)}(s|a) f_y^{(1)}(s,a,a')\right)}_{\bar{f}_y^{(2)}(a,a')} + \mathcal{O}\left(x^3\right)\right].\end{aligned} \tag{8}$$

Here we have defined $\bar{f}_y^{(1)}(a,a')$ and $\bar{f}_y^{(2)}(a,a')$ as first- and second-order averages of f.

We can then evaluate the transfer entropy rate (omitting higher order terms to fit the expression on one line):

$$
\begin{aligned}
\dot{T}_{s\to a} &= \sum_{s,a} p(s,a) \sum_{a'} w^{s}_{a'a} \ln \frac{w^{s}_{a'a}}{\bar{w}_{a'a}} \\
&= \sum_{s,a} \left[p^{(0)}_y(s,a) + x\, p^{(1)}_y(s,a)\right] \sum_{a'} w^{0}_{a'a} \left[1 + x\, f^{(1)}_y(s,a,a') + x^2\, f^{(2)}_y(s,a,a')\right] \ln \frac{\left[1 + x\, f^{(1)}_y(s,a,a') + x^2\, f^{(2)}_y(s,a,a')\right]}{\left[1 + x\, \bar{f}^{(1)}_y(a,a') + x^2\, \bar{f}^{(2)}_y(a,a')\right]} \\
&= \sum_{s,a} \left[p^{(0)}_y(s,a) + x\, p^{(1)}_y(s,a)\right] \sum_{a'} w^{0}_{a'a} \left[x \left(f^{(1)}_y(s,a,a') - \bar{f}^{(1)}_y(a,a')\right)\right. \\
&\quad \left. + \frac{1}{2} x^2 \left(\left[f^{(1)}_y(s,a,a') - \bar{f}^{(1)}_y(a,a')\right]^2 + 2\left[f^{(2)}_y(s,a,a') - \bar{f}^{(2)}_y(a,a')\right]\right)\right] \\
&= x \sum_{s,a,a'} w^{0}_{a'a} p^{(0)}_y(s,a) \left(f^{(1)}_y(s,a,a') - \bar{f}^{(1)}_y(a,a')\right) + x^2 \sum_{s,a,a'} w^{0}_{a'a} p^{(1)}_y(s,a) \left(f^{(1)}_y(s,a,a') - \bar{f}^{(1)}_y(a,a')\right) \\
&\quad + \frac{x^2}{2} \sum_{s,a,a'} w^{0}_{a'a} p^{(0)}_y(s,a) \left(\left[f^{(1)}_y(s,a,a') - \bar{f}^{(1)}_y(a,a')\right]^2 + 2\left[f^{(2)}_y(s,a,a') - \bar{f}^{(2)}_y(a,a')\right]\right) \\
&= x \sum_{a,a'} w^{0}_{a'a} v^{(0)}_y(a) \left(\underbrace{\sum_{s} p^{(0)}_y(s|a) f^{(1)}_y(s,a,a')}_{\bar{f}^{(1)}_y(a,a')} - \bar{f}^{(1)}_y(a,a')\right) \\
&\quad + x^2 \sum_{s,a,a'} w^{0}_{a'a} \left[p^{(1)}_y(s|a) v^{(0)}_y(a) + p^{(0)}_y(s|a) v^{(1)}_y(a)\right] \left(f^{(1)}_y(s,a,a') - \bar{f}^{(1)}_y(a,a')\right) \\
&\quad + \frac{x^2}{2} \sum_{s,a,a'} w^{0}_{a'a} p^{(0)}_y(s,a) \left(\left[f^{(1)}_y(s,a,a') - \bar{f}^{(1)}_y(a,a')\right]^2 + 2\left[f^{(2)}_y(s,a,a') - \bar{f}^{(2)}_y(a,a')\right]\right) \\
&= 0 + \frac{x^2}{2} \sum_{s,a,a'} w^{0}_{a'a} p^{(0)}_y(s,a) \left[f^{(1)}_y(s,a,a') - \bar{f}^{(1)}_y(a,a')\right]^2 + x^2 \sum_{a,a'} w^{0}_{a'a} v^{(1)}_y(a) \left(\underbrace{\sum_{s} p^{(0)}_y(s|a) f^{(1)}_y(s,a,a')}_{\bar{f}^{(1)}_y(a,a')} - \bar{f}^{(1)}_y(a,a')\right) \\
&\quad + x^2 \sum_{a,a'} w^{0}_{a'a} v^{(0)}_y(a) \left[\underbrace{\sum_{s} \left(p^{(0)}_y(s|a) f^{(2)}_y(s,a,a') + p^{(1)}_y(s|a) f^{(1)}_y(s,a,a')\right)}_{\bar{f}^{(2)}_y(a,a')} - \bar{f}^{(2)}_y(a,a')\right] \\
&= \frac{x^2}{2} \sum_{s,a,a'} w^{0}_{a'a} p^{(0)}_y(s,a) \left[f^{(1)}_y(s,a,a') - \bar{f}^{(1)}_y(a,a')\right]^2 + \mathcal{O}\left(x^3\right).
\end{aligned}
\tag{9}
$$

This rather tedious calculation shows that the transfer entropy rate $\dot{T}_{s\to a}$ is quadratic in x, and, moreover, if y is also small then the smallest possible term in an expansion around $(x,y) = (0,0)$ is $\mathcal{O}(x^2)$, with

$$
\dot{T}_{s\to a} = \frac{x^2}{2} \sum_{s,a,a'} w^{0}_{a'a} p^{(0)}_0(s,a) \left[f^{(1)}_0(s,a,a') - \bar{f}^{(1)}_0(a,a')\right]^2 + \mathcal{O}\left(x^3, x^2 y\right). \tag{10}
$$

By symmetry, the other transfer entropy rate is similarly quadratic in y^2, behaving as

$$
\dot{T}_{a\to s} = \frac{y^2}{2} \sum_{a,s,s'} w^{s's}_0 p^{(0)}_{x=0}(s,a) \left[g^{(1)}_0(a,s,s') - \bar{g}^{(1)}_0(s,s')\right]^2 + \mathcal{O}\left(y^3, y^2 x\right). \tag{11}
$$

B. Performance Metric

Consider now a family of performance metrics that generally quantify how consistently the chemotaxing agent chooses the right action in the face of a given stimulus. We formalize this metric using a "score function" $q(s, a)$, which returns a scalar score for each s and a pair. The performance metric is then

$$\Delta \equiv \sum_{s,a} q(s, a)p(s, a). \tag{12}$$

We further assume that the stimulus variable s has some structure such that each s has an inverse $-s$, and that the score function is antisymmetric, satisfying $q(s, a) = -q(-s, a)$. This class of performance metrics includes the chemotactic index, where

$$q_{\text{c.i.}} = \begin{cases} 1, & s = u,\, a = R, \\ -1, & s = d,\, a = R, \\ 0 & a = T. \end{cases} \tag{13}$$

Using the definition of the inverse for s, it is clear from Eq. (3) that when $x = 0$ we have $w^{s}_{a'a} = w^{-s}_{a'a}$ and $w^{s's}_{a} = w^{-s'-s}_{a}$. Thus since the transition rates are invariant under the inverse operation, the steady-state probability must be as well such that $p_{0,y}(s, a) = p_{0,y}(-s, a)$ when $x = 0$.

Now consider the performance metric $\Delta(x, y)$ as a function of x and y. When $x = 0$, using the symmetries and antisymmetries discussed above, this is

$$\begin{aligned} \Delta(0, y) &= \sum_{s,a} q(s, a)p_{0,y}(s, a) \\ &= \sum_{s,a} q(-s, a)p_{0,y}(-s, a) \\ &= -\sum_{s,a} q(s, a)p_{0,y}(s, a) \\ &= -\Delta(0, y). \end{aligned} \tag{14}$$

Since Δ satisfies this property, there can be no surviving y terms when $x = 0$. It thus follows that the performance metric must have an expansion around $(x, y) = (0, 0)$ of the form

$$\Delta = D_1 x + D_{11} xy + D_2 x^2 + \mathcal{O}\left(x^3 + x^2 y + xy^2\right). \tag{15}$$

C. Final Argument

Above, we have shown that around $(x, y) = (0, 0)$ the transfer entropy rates must scale as

$$\dot{T}_{s\to a} = C_{sa} x^2 + \mathcal{O}(x^3, x^2 y), \tag{16a}$$

$$\dot{T}_{a\to s} = C_{as} y^2 + \mathcal{O}(y^3, y^2 x), \tag{16b}$$

while the performance metric has expansion

$$\Delta = D_1 x + D_{11} xy + D_2 x^2 + \mathcal{O}\left(x^3 + x^2 y + xy^2\right). \tag{17}$$

Thus, near $(x, y) = (0, 0)$, where transition rates between states of one degree of freedom are only weakly dependent on the state of the other, we must have

$$\boxed{\Delta \approx A\sqrt{\dot{T}_{s\to a}} + B\sqrt{\dot{T}_{s\to a}\dot{T}_{a\to s}} + C\,\dot{T}_{s\to a}.} \tag{18}$$

This concludes the proof.

II. FULL-HISTORY TRANSFER ENTROPY RATES

Here we derive semi-analytic expressions for the full-history transfer entropy rates, which will then allow us to compute them numerically. We consider a 4-state bipartite system with states x and y, which can each take two states labeled 0 and 1, and focus on the steady state. We use the notation $\{x\}_{-\infty}^{t}$ to indicate the full-history trajectory of x up to time t.

Recall that the full-history transfer entropy rates are defined as

$$\dot{\mathcal{T}}_{x\to y} \equiv \lim_{\mathrm{d}t\to 0} \frac{I[y_{t+\mathrm{d}t}; \{x\}_{-\infty}^{t} | \{y\}_{-\infty}^{t}]}{\mathrm{d}t}. \tag{19}$$

Evaluating the conditional mutual information gives

$$\dot{\mathcal{T}}_{x\to y} = \lim_{\mathrm{d}t\to 0} \frac{1}{\mathrm{d}t} \left\langle \left\langle \ln \frac{p\left(y_{t+\mathrm{d}t}|\{x\}_{-\infty}^{t}, \{y\}_{-\infty}^{t}\right)}{p\left(y_{t+\mathrm{d}t}|\{y\}_{-\infty}^{t}\right)} \right\rangle_{p\left(y_{t+\mathrm{d}t}|\{x\}_{-\infty}^{t}, \{y\}_{-\infty}^{t}\right)} \right\rangle_{p\left(\{x\}_{-\infty}^{t}, \{y\}_{-\infty}^{t}\right)}. \tag{20}$$

The conditional probability in the numerator is straightforward to evaluate, simply given by $p\left(y_{t+\mathrm{d}t} = 1 - y_t|\{x\}_{-\infty}^{t}, \{y\}_{-\infty}^{t}\right) = w_{(1-y_t)y_t}^{x_t}\mathrm{d}t$ since the distribution of $y_{t+\mathrm{d}t}$ is entirely determined by the current states y_t and x_t and thus $p\left(y_{t+\mathrm{d}t}|\{x\}_{-\infty}^{t}, \{y\}_{-\infty}^{t}\right) = p\left(y_{t+\mathrm{d}t}|x_t, y_t\right)$. The conditional probability in the denominator, by contrast, is more difficult to evaluate. It can be expanded as

$$p\left(y_{t+\mathrm{d}t}|\{y\}_{-\infty}^{t}\right) = \left\langle p\left(y_{t+\mathrm{d}t}|\{x\}_{-\infty}^{t}, \{y\}_{-\infty}^{t}\right)\right\rangle_{p\left(\{x\}_{-\infty}^{t}|\{y\}_{-\infty}^{t}\right)} = \left\langle p\left(y_{t+\mathrm{d}t}|x_t, y_t\right)\right\rangle_{p\left(x_t|\{y\}_{-\infty}^{t}\right)}, \tag{21}$$

again using $p\left(y_{t+\mathrm{d}t}|\{x\}_{-\infty}^{t}, \{y\}_{-\infty}^{t}\right) = p\left(y_{t+\mathrm{d}t}|x_t, y_t\right)$. This, in turn, requires evaluating the conditional probability $q_t \equiv p\left(x_t = 1|\{y\}_{-\infty}^{t}\right)$, which we call the "belief" about the state of x_t given the history of y. We will evaluate this belief by considering its dynamics. To do this, it will be useful to define the expected rate of y state changes given the belief about the x state, as

$$\hat{w}_{y_t}(q_t) \equiv (1 - q_t)w_{(1-y_t)y_t}^{0} + q_t w_{(1-y_t)y_t}^{1}. \tag{22}$$

Here we have simply averaged the transition rate from y_t to $1 - y_t$ over the distribution q_t.

First, we consider the evolution of the probability q_t over a time interval $\mathrm{d}t$ where y remains constant. Note that y remains constant with probability $1 - \hat{w}_{y_t}(q_t)\mathrm{d}t$. Likewise, the probability of state $x_{t+\mathrm{d}t} = 1$ and no change in y given the known history of y obeys, to first order in $\mathrm{d}t$,

$$p\left(x_{t+\mathrm{d}t} = 1, \text{no change in } y|\{y\}_{-\infty}^{t}\right) = q_t(1 - w_{(1-y_t)y_t}^{1}\mathrm{d}t - w_{y_t}^{01}\mathrm{d}t) + (1 - q_t)w_{y_t}^{10}\mathrm{d}t. \tag{23}$$

Here we use the fact that either two changes of the x state, or a change of both the x and y states, would only occur with probability second-order in $\mathrm{d}t$. The probability $q_{t+\mathrm{d}t}$, given no change in y, will then be

$$\begin{aligned} q_{t+\mathrm{d}t} = p\left(x_{t+\mathrm{d}t} = 1|\{y\}_{-\infty}^{t}, \text{no change in } y\right) &= \frac{p\left(x_{t+\mathrm{d}t} = 1, \text{no change in } y|\{y\}_{-\infty}^{t}\right)}{p\left(\text{no change in } y\right)} \\ &= \frac{q_t(1 - w_{(1-y_t)y_t}^{1}\mathrm{d}t - w_{y_t}^{01}\mathrm{d}t) + (1 - q_t)w_{y_t}^{10}\mathrm{d}t}{1 - \hat{w}_{y_t}(q_t)\mathrm{d}t}. \end{aligned} \tag{24}$$

Expanding the fraction to first order in $\mathrm{d}t$, subtracting q_t, dividing by $\mathrm{d}t$, and defining $\dot{q}_t = \lim_{\mathrm{d}t\to 0}(q_{t+\mathrm{d}t} - q_t)/\mathrm{d}t$, gives the evolution of q_t given no change in y:

$$\begin{aligned} \dot{q}_t &= (1 - q_t)w_{y_t}^{10} + q_t(\hat{w}_{y_t}(q_t) - w_{(1-y_t)y_t}^{1} - w_{y_t}^{01}) \\ &= (1 - q_t)w_{y_t}^{10} + q_t(1 - q_t)(w_{(1-y_t)y_t}^{0} - w_{(1-y_t)y_t}^{1}) - q_t w_{y_t}^{01}. \end{aligned} \tag{25}$$

Here in the second term we used the definition of $\hat{w}_{y_t}(q_t)$.

Second, we consider the evolution of the probability q_t given an instantaneous change in y at time t. Let y_t^- and y_t^+ be the pre- and post-jump values of y, and let q_t^- and q_t^+ be the pre- and post-jump conditional probabilities of $x_t = 1$. Since this is an instantaneous change in y, x must be constant, so we can use Bayes' rule to write

$$q_t^+ = \frac{q_t^- w_{(1-y_t^-)y_t^-}^{1}\mathrm{d}t}{q_t^- w_{(1-y_t^-)y_t^-}^{1}\mathrm{d}t + (1 - q_t^-)w_{(1-y_t^-)y_t^-}^{0}\mathrm{d}t} = \frac{q_t^- w_{(1-y_t^-)y_t^-}^{1}}{\hat{w}_{y_t^-}(q_t^-)}. \tag{26}$$

Thus the random variable q_t evolves through deterministic continuous dynamics, except at time-points where y stochastically changes state, and thus q_t jumps.

We can write the full dynamics of the distribution $p(q_t)$ as a single partial differential equation. Defining the deterministic drift force

$$F_{y_t}(q_t) \equiv (1-q_t)w^{10}_{y_t} + q_t(1-q_t)(w^0_{(1-y_t)y_t} - w^1_{(1-y_t)y_t}) - q_t w^{01}_{y_t}, \tag{27}$$

the jump map

$$J_{y_t^-}(q_t^-) = \frac{q_t^- w^1_{(1-y_t^-)y_t^-}}{\hat{w}_{y_t^-}(q_t^-)}, \tag{28}$$

and the probability $\rho_y(q)$ as the joint probability of having belief q and current state y, we have

$$\frac{\partial \rho_y(q)}{\partial t} = -\frac{\partial}{\partial q}\left[F_{y_t}\rho_y(q)\right] - \hat{w}_y(q)\rho_y(q) + \int \mathrm{d}q'\, \hat{w}_{(1-y)}(q')\,\rho_{(1-y)}(q')\delta(q - J_{1-y}(q')), \tag{29}$$

for Dirac-delta function $\delta(\cdot)$. We can simplify this PDE by evaluating the integral on the right-hand side, using the delta function identity $\delta(g(x)) = \delta(x-x_0)/|g'(x_0)|$, for a function $g(x)$ which has a unique zero at x_0. Applying this identity gives

$$\begin{aligned} \int \mathrm{d}q'\, \hat{w}_{(1-y)}(q')\,\rho_{(1-y)}(q')\delta(q - J_{1-y}(q')) &= \int \mathrm{d}q'\, \hat{w}_{(1-y)}(q')\,\rho_{(1-y)}(q') \frac{\delta\left(q' - J^{-1}_{(1-y)}(q)\right)}{J'_{(1-y)}\left(J^{-1}_{(1-y)}(q)\right)} \\ &= \frac{\hat{w}_{(1-y)}\left(J^{-1}_{(1-y)}(q)\right)\,\rho_{(1-y)}\left(J^{-1}_{(1-y)}(q)\right)}{J'_{(1-y)}\left(J^{-1}_{(1-y)}(q)\right)}, \end{aligned} \tag{30}$$

where $J^{-1}_{(1-y)}$ denotes the inverse of $J_{(1-y)}$.

At steady state, $\partial\rho_y(q)/\partial t = 0$, and thus $\rho_y(q)$ must solve

$$\frac{\partial}{\partial q}\left[F_{y_t}\rho_y(q)\right] = -\hat{w}_y(q)\rho_y(q) + \frac{\hat{w}_{(1-y)}\left(J^{-1}_{(1-y)}(q)\right)\,\rho_{(1-y)}\left(J^{-1}_{(1-y)}(q)\right)}{J'_{(1-y)}\left(J^{-1}_{(1-y)}(q)\right)} \tag{31}$$

for each of $y=0$ and $y=1$, together with normalization

$$\sum_{y=\{0,1\}} \int \mathrm{d}q\, \rho_y(q) = 1. \tag{32}$$

This must be solved numerically.

Armed with the distribution $\rho_y(q)$, we are now prepared to evaluate the full-history transfer entropy rate:

$$\begin{aligned} \dot{\mathcal{T}}_{x\to y} &= \lim_{\mathrm{d}t\to 0}\frac{1}{\mathrm{d}t}\left\langle\left\langle \ln\frac{p\left(y_{t+\mathrm{d}t}|\{x\}^t_{-\infty},\{y\}^t_{-\infty}\right)}{p\left(y_{t+\mathrm{d}t}|\{y\}^t_{-\infty}\right)}\right\rangle_{p\left(y_{t+\mathrm{d}t}|\{x\}^t_{-\infty},\{y\}^t_{-\infty}\right)}\right\rangle_{p\left(\{x\}^t_{-\infty},\{y\}^t_{-\infty}\right)} \\ &= \left\langle (1-q)w^0_{(1-y)y}\ln\frac{w^0_{(1-y)y}}{\hat{w}_y(q)} + q w^1_{(1-y)y}\ln\frac{w^1_{(1-y)y}}{\hat{w}_y(q)}\right\rangle_{\rho_y(q)}. \end{aligned} \tag{33}$$

Once the distribution $\rho_y(q)$ has been computed numerically, this average is in turn straightforward to compute.
